# Transcribed Ultraconserved Regions: New regulators in cancer signaling and potential biomarkers

**DOI:** 10.1590/1678-4685-GMB-2022-0125

**Published:** 2023-01-09

**Authors:** Jaqueline Carvalho de Oliveira

**Affiliations:** 1 Universidade Federal do Paraná, Departamento de Genética, Curitiba, Paraná, Brazil.

**Keywords:** T-UCR, apoptosis, proliferation, metastasis, prognosis

## Abstract

The ultraconserved regions (UCRs) are 481 genomic elements, longer than 200 bp, 100% conserved in human, mouse, and rat genomes. Usually, coding regions are more conserved, but more than 80% of UCRs are either intergenic or intronic, and many of them produce long non-coding RNAs (lncRNAs). Recently, the deregulated expression of transcribed UCRs (T-UCRs) has been associated with pathological conditions. But, differently from many lncRNAs with recognized crucial effects on malignant cell processes, the role of T-UCRs in the control of cancer cell networks is understudied. Furthermore, the potential utility of these molecules as molecular markers is not clear. Based on this information, the present review aims to organize information about T-UCRs with either oncogenic or tumor suppressor role associated with cancer cell signaling, and better describe T-UCRs with potential utility as prognosis markers. Out of 481 T-UCRs, 297 present differential expression in cancer samples, 23 molecules are associated with tumorigenesis processes, and 12 have more clear potential utility as prognosis markers. In conclusion, T-UCRs are deregulated in several tumor types, highlighted as important molecules in cancer networks, and with potential utility as prognosis markers, although further investigation for translational medicine is still needed.

## Introduction

The ultraconserved regions (UCRs) are 481 genomic elements longer than 200 bp (range: 200-779 bp) that are absolutely conserved among orthologous regions of human, mouse, and rat genomes. These regions also exhibit extremely high levels of conservation in other species, such as fish, chicken, and fugu, strongly suggesting an extreme negative selection of these sequences (Bejerano et al., 2004). The UCRs were computationally identified in 2004, by Bejerano *et al*. (2004) and they are widely distributed on all human chromosomes, except on chromosomes 21 and Y. 

**Figure 1 -:**
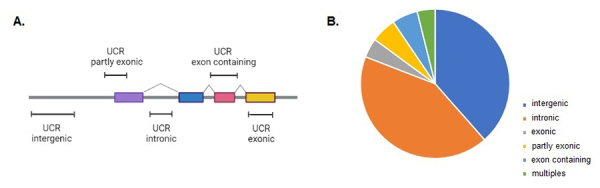
UCR classification according genomic location. A. UCR types according the position and closest coding gene. B. Percentage of UCR types. Data based on Mestdagh et al. (2010) re-annotation.

Annotating all UCR sequences using the genome build hg18 and matching their location to the human RefSeq genes, Mestdagh et al. (2010) organized the UCRs into five different categories: 38.7% UCRs were intergenic; 42.6% were intronic; 4.2% exonic; 5% partly exonic; and 5.6% were exon containing. For 3.9%, the genomic annotation varies as a result of of host gene splice variants, and these UCRs are categorized as “multiple” (Mestdagh *et al*., 2010) (Figure 1A).

Usually, coding regions are more conserved than noncoding regions, but it is interesting to highlight that more than 80% of UCRs are intergenic or intronic (Figure 1B). Additionally, among the intronic UCRs, almost 58% were detected in the antisense orientation compared with the host gene, suggesting that most of these molecules did not represent only intronic transcription of the known host genes (Calin et al., 2007). These numbers indicate that most T-UCRs may be lncRNAs (long noncoding RNAs), defined as RNAs larger than 200 bp, mostly without coding potential. 

The genome-wide profiling reveals that most UCRs are transcriptional active; therefore, these regions are also named transcribed UCRs (T-UCRs). About 34% of UCRs were detected in all 19 normal tissue samples analyzed, and 93% of the UCRs were expressed in at least one tissue type (Calin et al., 2007). 

The first association between T-UCRs and cancer investigated the expression of the 481 sequences in tumor samples, including chronic lymphocytic leukemia (CLL), colorectal carcinoma (CRC), and hepatocellular carcinoma (HCC) patients, as well as corresponding non- tumor tissues. Hierarchical clustering differentiated each tumor type from others and their normal counterparts, showing specific groups of T-UCRs differentially expressed in tumor types (Calin et al., 2007). Since then, the T-UCR deregulated expression has been associated with several tumor types and pathological conditions (Fabris and Calin, 2017; Pereira Zambalde et al., 2020). 

During carcinogenesis, it is well known that normal cells evolve to a neoplastic state, thus altering a basic mechanism that include sustained proliferative signaling, loss of growth suppressors, apoptosis resistance, invasion and metastasis activation, among other common characteristics (Hanahan, 2022). LncRNAs exert crucial effects on malignant cell processes, including influence on proliferation and apoptosis rates (de Oliveira et al., 2019), but little is known about but the role of T-UCRs in controlling cellular processes. Furthermore, most studies about T-UCR in cancer are focused on differential expression in tumor samples, but the potential utility of these molecules as molecular markers is not clear.

Based on this information, the goal of the present review is to organize information about T-UCRs associated with cancer cell signaling, and better describe describe T-UCRs with potential utility as diagnosis and prognosis markers in different tumor types. 

Cancer cell mechanisms influenced by T-UCR

Most T-UCRs associated with cancer show deregulated expression levels in tumor samples, presenting expression analysis but not functional assays. However, many recent studies have also demonstrated T-UCRs influencing hallmarks of cancer. Herein, I organized T-UCRs with highlighted influence on cancer cell mechanisms, focused on networks and the molecular details. 

T-UCRs with oncogenic role

After modulation, many studies evaluate T-UCR effects on proliferation, apoptosis, and migration/invasion in cancer cell lines. For example, silencing of Uc.73 in CRC cell lines reduced proliferation and increased apoptosis levels (Calin et al., 2007). Also, Uc.147, an important oncogenic T-UCR highlighted in luminal breast cancer (BC) cells, influenced cell viability, colony formation, cell cycle dynamics, and apoptosis rates (Pereira Zambalde et al., 2021). In pancreatic cells (PC), Uc.190, Uc.233, and Uc.270 induced proliferation rates (Jiang et al., 2016). 

**Figure 2 -:**
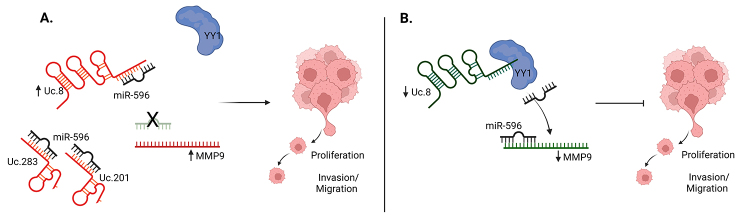
Mechanism of action of Uc.8, Uc.201, and Uc.283. A. In cancer cells, Uc.8, Uc.201, and Uc.283 high expression induce invasion/migration and proliferation. B. Uc.8 silencing decreased invasion/migration and proliferation. Green molecules meaning low expression and red meaning high expression. Created with BioRender.com

Focused on T-UCRs with molecular mechanisms better characterized, Uc.8 is an exciting example. Containing 2,435 nucleotides (including the 216-nt ultraconserved sequence), the Uc.8 transcript is located within intron 1 of CASZ1, a zinc-finger transcription factor, but it is expressed independently of the host gene (Olivieri et al., 2016). In bladder cancer (BlC) cells, Uc.8 silencing decreased invasion/migration and proliferation by interacting with miR-596, and preventing this miR from interacting with its target MMP9 (Olivieri *et al*., 2016). Additionally, the polycomb protein Yin Yang 1 (YY1) is a binding mediator between miR-596 and Uc.8. It was suggested that the bind of YY1 on Uc.8 may change its conformation, inhibiting miR-596/Uc.8 interaction (Terreri et al., 2016) (Figure 2).

Uc.8 is a T-UCR that may be found initially in cytoplasm and the nucleus. Interestingly, the analysis of subcellular localization of this T-UCR may be a potential prognostic biomarker for BlC, since the transcript was found more prevalent in cytoplasmic localization in high-grade samples (Terreri et al., 2021). It is relevant to highlight that the interaction Uc.8/miR-596 is found essentially in the cytoplasm, being a mechanism potentially associated with this more aggressive phenotype. 

The miR-596 has been described as a tumor suppressor miR in several tumor types, previously associated with Wnt/β-catenin signaling (Wei et al., 2019; Dai et al., 2021), Smurf1/p53 (Ma et al., 2017) and IGF2BP2 expression (Fen et al., 2020). 

The miR-596, associated with Uc.8, also has the predicted binding site of other eight T-UCRs (Uc.195, Uc.201, Uc.283, Uc.305, Uc.388, Uc.390, Uc.393, Uc.457). Through the miR-596 modulation *in vitro*, it was suggested that Uc.283 may be a target of miR-596 while Uc.201 may acts as a sponge in combination with Uc.8 to repress miR-596 expression (Terreri et al., 2016) (Figure 2).

It was found that Uc.51 was highly expressed in BC tissues and cell lines, promoting cell proliferation, migration, and both in vitro and in vivo invasion (Olivieri et al., 2016; Shi et al., 2021). Uc.51 can interact with NONO protein (Non-POU domain-containing octamer-binding protein), maintaining its stability and activating the phosphorylation of CREB (Shi *et al*., 2021). 

NONO protein interacts with DNA, RNA, and multiple proteins. It participates in various biological processes, such as DNA damage repair, pre-mRNA splicing, transcriptional regulation, nuclear RNA retention, in addition to being associated with several tumor types (Feng et al., 2020). Previous studies have demonstrated that NONO is necessary for cAMP-dependent activation of CAMP response element-binding proteins (CREB) target genes (Amelio et al., 2007), an important pathway in several tumor types, including BC (Ma et al., 2019; Feng *et al*., 2020). Several lncRNAs have been associated with NONO protein (Ma *et al*., 2019; Chen *et al*., 2020), and the association with Uc.51 exemplify that this protein may be an important interaction with UCRs. 

One T-UCR with an important role in different types of cancer and related to many cell processes is the Uc.63. Associated with BC, BlC, PC, and gastric cancer (GC), Uc.63 has an oncogenic role, independently of the host *XPO1* gene, inducing proliferation and cell migration, also decreasing apoptosis levels in BC, BlC cell lines (Sekino et al., 2019). 

In PC cell lines, the Uc.63 sequence has binding sites to miR-130b, and the expression of this miR was disturbed by Uc.63 modulation, which also affects the MMP2 miR target (Sekino et al., 2017). *In vitro* modulation of Uc.63 also repressed GC cell growth and migration via NF-κB signaling (Sakamoto et al., 2020). In more detail, the Uc.63 expression induced the expression of p65, which is one of the important subunits in the NF-κB complex. Additionally, silencing of RELA (which is the coding gene of p65) was able to reduce the effect of Uc.63 induced expression in cell growth (Sakamoto *et al*., 2020). 

The androgen receptor (AR), an important protein in several tumor types including prostate, breast, and bladder (Solomon et al., 2019; Tripathi and Gupta, 2020) and correlated with the Uc.63 expression; had its expression disrupted by Uc.63 modulation in BlC cells. Additionally, the knockdown of Uc.63 increased sensitivity to Cisplatin chemotherapy in regular UMUC3 positive cells and also re-sensitized the UMUC3-Cisplatin resistant cells. On the other hand, an overexpression of Uc.63 did not affect Cisplatin sensitivity in AR-negative cells (Marini et al., 2017; Sekino et al., 2017; Sakamoto et al., 2020). 

Uc.83 was also associated with induced cell growth (Vannini et al., 2022). Uc.83 has 1143-bp and is mapped within a lncRNA, the LINC01876, but expressed independently of the host. In functional analysis, Uc.83-silencing decreased cell growth while the up-regulation increased cell proliferation, partially mediated by the phosphorylation of AKT and ERK 1/2, two important biomarkers of lung cancer cell proliferation (Vannini *et al*., 2022). 

Another important T-UCR is the TRA2β4 mRNA isoform containing Uc.138. The human TRA2B gene contains 10 exons, and it produces five mRNA isoforms (TRA2β1 to 5) (Nayler et al., 1998). The functional Tra2β protein is translated from TRA2β1 mRNA and it produces a nuclear protein that plays the role of a sequence-specific pre-mRNA splicing enhancer (Tsuda et al., 2011). TRA2β1 lacking exon 2 is a region that encodes multiple premature termination codons; on the other hand, TRA2β4 isoform contains exon 2, it is nuclear, and it does not translate to a functional protein. Interestingly, Uc.138 (with 419-bp) spans the exon 2 (276 bp) and its neighboring introns, and only TRA2β4 isoform contains a complete version of exon 2 (Nayler *et al*., 1998).

This T-UCR is preferentially expressed in colon cancer cells and acts in proliferation control by interaction with the nucleolin protein (Satake et al., 2018). Nucleolin is a multifunctional protein acts by modulating rDNA transcription, RNA metabolism, and ribosome assembly, with expression and localization that is abnormal in tumors, affecting proliferation, survival, and metastasis of cancer cells (Chen and Xu, 2016). 

Additionally, specific TRA2β4 siRNA did not change TRA2β1 mRNA or Tra2β protein levels and it inhibited cell growth in HCC cell line by senescence, not affecting apoptosis levels. TRA2β4 may sequester Sp1 from occupying promoters of target genes, including CDKN1A, leading to cell growth by interrupting the senescence-related gene expression program (Kajita et al., 2016).

In a recent study of HCC cells, the overexpression of TRA2B4 or exon 2 increased the percentage of G2/M cells and deregulated expression of cell-cycle related gene in a concordant manner. After 5-fluorouracil or Adriamycin treatment, the overexpression of TRA2B4 increased viability, associating this T-UCR also with drug resistance (Kuwano et al., 2021). Additionally, increased migration, and enhanced tumorigenesis *in vivo* after overexpression reinforcing the point that Uc.138/TRA2β4 transcript plays an oncogenic role in tumor progression (Kuwano *et al*., 2021). It is interesting to note that Uc.138 contains a stem-loop structure (449-488 nt) and the introduction of mutations in the stem-loop motif canceled these effects, thus showing the importance of UCR sequence in TRA2β4 transcript role (Kuwano *et al*., 2021).

**Figure 3 -:**
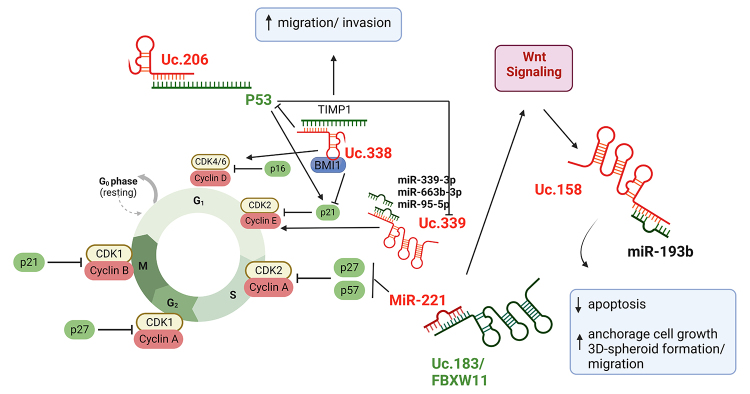
Mechanism of action of Uc.158, Uc.183, Uc.206, Uc.338, and Uc.339. Green molecules meaning low expression and red meaning high expression. Created with BioRender.com

Aiming to better understand T-UCR mechanisms in liver cancer, Carotenuto et al. (2017) studied molecules regulated in Wnt/β-catenin signaling network, one of the major genetic pathway deregulated in cancer. With animal models (hypomorphic Apc mice that developed Wnt/β-catenin dependent HCC), overexpression of Uc.158 was found in Wnt/β-catenin dependent HCC compared to normal liver or β-catenin negative-induced HCC, and this T-UCR were reduced after treatment with Wnt/β-catenin inhibitors. Silencing of Uc.158 increased apoptosis and reduced anchorage cell growth, 3D-spheroid formation, and spheroid-based cell migration in HepG2 and SW1 cells, also associated with miR-193b presence. A high expression of Uc.158 was also found in cholangiocarcinoma patients (Carotenuto *et al*., 2017) (Figure 3).

Also associated with major regulators in cancer, the high expression of Uc.206 in cervical cancer negatively regulates p53 expression by speciﬁc target of 3’ untranslated region (3’UTR) of mRNA, affecting cervical cell proliferation and apoptosis levels (Li et al., 2017). Uc.338 and Uc.339 are also associated with p53 regulation (Figure 3).

Uc.338 is a 590-pb RNA T-UCR that influences important mechanism in several cancer types. For example, in CRC and HCC, Uc.338 induced proliferation and cell cycle G1/S transition, causing cell migration and invasion in LC and CC (Braconi et al., 2011; Bo et al., 2016; Gao et al., 2016; Li et al., 2017; Zhang et al., 2018). Uc.338 is located within *PCBP2* (the poly(rC) binding protein 2) gene but transcribed independently of the host. About mechanisms, Uc.338 induced proliferation and cell cycle G1/S transition via PI3K/AKT pathway, possibly targeting p21 down-regulation and cyclin D1 up-regulation (Zhang *et al*., 2018) (Figure 3). Additionally, silencing of this T-UCR, in CC cells, inhibited cell migration and invasion by TIMP1 direct regulation (Li *et al*., 2017). 

Uc.338 promotes proliferation and induces cell cycle progression in HCC cells associated with BMI1, modulating the transcription of *CDKN1A* and partially repressing p21 (Bo et al., 2016). To better understand the mechanism of action, Wen et al. (2018) performed chromatin isolation by RNA purification followed by mass spectrometry and genomic analysis to identify Uc.338-binding proteins and occupancy sites throughout the genome. The genomic region of Uc.338 occupancy was enriched in binding motifs homologous to the tumor suppressors Pax6 and p53. Interestingly, after Uc.338 knockdown, an almost 30% increase in p53 activity was observed. Additionally, it was identified around 400 potential target genes with Uc.338-binding sites within 9 kb of gene loci were identified, many of them involved in cell proliferation. Furthermore, the plasminogen activator inhibitor-1 RNA-binding protein (PAI-RBP1) was identified as a Uc.338 RNA-binding partner (Wen *et al*., 2018).

In a close genome region, Uc.339 is overexpressed in HCC cells and HCC-derived exosomes, contributing to a pro-tumoral HCC microenvironment (Kogure et al., 2013). The Uc.339 is directly regulated by P53. Its acts as a decoy for miR-339-3p, -663b-3p, and -95-5p, able to up-regulate Cyclin E2, a direct target of all these microRNAs (miRs), and promote cancer growth and migration (Figure 3). Interestingly, *in vitro* modulation of these miRs do not affect Uc.339 levels, acting as a type of “entrapping” (Vannini et al., 2017).

Uc.339 expression induces an increase in viability, migration in accordance with the silencing of this molecule, recusing the percentage of cells in the S-phase and increasing cellular apoptosis (Vannini et al., 2017).

The last T-UCR described herein with an oncogenic role is Uc.416. This molecule was found to be overexpressed in gastric cancer (GC) and affects cancer growth and migration (Sekino et al., 2018). It was demonstrated that Uc.416 is associated with cell growth by regulating insulin-like growth factor-binding protein 6 (IGFBP6). Additionally, this T-UCR has miR-153 predicted binding site and, in GC cell lines, modulation of this miR was able to disturb Uc.416 expression (Goto et al., 2016).

Furthermore, Uc.416 reduced cell growth and cell migration activity related to the expression of SNAI1, VIM and inversely associated with the expression of CDH1 and miR-153 (Sekino et al., 2018).

T-UCRs with tumor suppressor role

Focused on T-UCRs with tumor suppressor role in cancer, Uc.38 was found down-regulated in breast cancer tissues and cell lines (Zhang et al., 2017; Zadrożna-Nowak et al., 2022). *In vitro*, Uc.38 overexpression inhibits cell growth and induces apoptosis by affecting PBX1, an important transcription factor important in development, and found deregulated in cancer cells (Zhang *et al*., 2017). 

Aiming to find novel T-UCRs involved in cell cycle regulation, Corrà et al. (2021) performed a genome-wide study, and presented 13 T-UCRs mutually exclusive with miR-221, a critical miR associated with G1/S transition by targeting cyclin-dependent kinase inhibitors, p27 and p57. Uc.96, Uc.110, and Uc.183 were the most effective in modulating cell cycle phases after silencing. Furthermore, Uc.183 was suggested as the best candidate to be negatively regulated by and interfere with miR-221 expression, affecting S phase of cell cycle (Corrà *et al*., 2021) (Figure 3). 

Uc.183 is localized on a *FBXW11* coding exon. The siRNAs designed against Uc.183 sequence, also affected both genes, so Uc.183/FBXW11 could be the same transcript. FBXW11 (F-box and WD repeat domain containing 11) is a vital protein acting by phosphorylation-dependent ubiquitination, associated with cell proliferation by targeting multiple substrates for degradation. Recently, FBXW11 mRNA was also demonstrated as a directed target of miR-221, associated with proliferation and apoptosis in osteosarcoma (OS), regulating Wnt signaling (Zhang et al., 2021).

Uc.454 has also been associated with tumor suppressor activity. Further, the low expression in the LC tumor tissues than that of adjacent non-tumor, by induced and silencing expression in lung cells, Uc.454 has been associated with low proliferation, low colony formation, decreased tumorigenesis *in vivo*, and high apoptosis level. 

The HSPA12B gene is located directly downstream of Uc.454, and this T-UCR has binding sites on 3’UTR in mRNA. It was demonstrated that Uc.454 decreased HSPA12B expression directly at transcriptional and translational level and Uc.454 is dependent of HSPA12B presence. Furthermore, the induced high expression of Uc.454 also inhibited cell migration and invasion by targeting K-Ras gene, and down-regulating P63 and MMP9 proteins (Zhou et al., 2018a, b)

T-UCRs with opposite influence in distinct tumor types 

Uc.160 is an important T-UCR highlighted in glioma, CRC and GC cells but with opposite influence in these tumor types. In CRC, Uc.160 expression was associated with increased proliferation and rates of motility (Honma et al., 2017; Kottorou et al., 2018). On the other hand, Uc.160 expression in GC cells reduced viability and proliferation *in vitro* and *in vivo*; furthermore induced apoptosis rates (Pang et al., 2018). 

The induced expression of Uc.160 reduced GC cell proliferation *in vitro* and *in vivo* (Pang et al., 2018) partially by inhibiting the phosphorylation of Akt and increasing PTEN expression, an important tumor suppressor protein (Honma et al., 2017; Pang *et al*., 2018). Showing the multiple pathways associated with Uc.160 and sometimes controversial, Pang and colleagues also demonstrated, in GC cells, the influence of miR-155 in Uc.160 expression in GC cells.

Mir-155 has a tumor suppressor role in GC cell lines and, previously, interaction between miR-155 and Uc.160 was suggested in the CLL (Calin et al., 2007). In GC cells, it was demonstrated that induced expression of miR-155 directly decreases Uc.160 expression. As both molecules have tumor suppressor activity in GC cells, an inverse correlation will be expected, different from the results presented and showing the complexity of Uc.160 mechanism of action (Pang et al., 2018). 

In glioma cells, Uc.160 is epigenetically silenced, apparently with more tumor suppressor activity. Uc.160 interacts with primary microRNA of the miR-376 cluster, positively regulating mature sequences and affecting the downstream miR-376-regulated genes, such as RING1, RYBP, and FOXP2. Many T-UCRs are described as negative regulators of miR expression, but in this example, Uc.160 shows interaction with primary microRNA (pri‐miRNA) molecule and acts as a positive regulator of cleavage, enhancing A‐to‐I editing on its mature sequence (Soler et al., 2022). 

Circulating RNAs, drug resistance and hypoxia

Circulating T-UCRs may also play an important role in the cancer process, for example, exosomes with Uc.189 from ESCC patients promoted proliferation, migration, and tube formation in human lymphatic endothelial cells. Mechanistically, Uc.189 regulated *EPHA2* expression by directly binding to its 3’UTR region (Ding et al., 2021). 

Related to drug resistance, Uc.160, Uc.283, and Uc.346 were found to be low expressed in 5-fluorouracil-resistant CRC cells, and both Uc.283 and Uc.346 were reduced in oxaliplatin-resistant cells (Kottorou et al., 2020). Furthermore, Uc.287 was found to be induced by synthetic androgen in PC (Hudson et al., 2013), Uc.300 was reduced, and Uc.324 was induced following all-trans-retinoic acid in neuroblastoma. *In vitro*, Uc.300 silencing also decreased the proliferation and invasiveness of ATRA-responsive cell lines (Watters et al., 2013).

Some T-UCRs were associated with cancer mechanisms only in specific conditions, for example, in hypoxia conditions. T-UCRs Uc.63, Uc.73, Uc.106, Uc.134, and Uc.475, previously associated with CRC, were induced more than two-fold after hypoxia and DMOG exposures (a widely used hypoxia mimetic) (Ferdin et al., 2013). Furthermore, Uc.475 down-regulation in HT-29 cells significantly decreased cell proliferation by G2/M arrest, but under normoxic conditions, this effect was not observed (Ferdin *et al*., 2013). 

T-UCRs as molecular diagnosis/prognosis markers

T-UCRs have been described as differentially expressed in several tumor types. Most studies only described a list of up or down expressions and, in this situation, this could be useful as a diagnosis marker. But simple diagnosis in cancer has limited application for new molecular markers, while prognosis markers able to identify patients with poor survival time or resistance to specific treatment have o huge interest for translation in patients’ medical conduct. 

In the following topics, I highlight the molecules associated with clinical features and with potential utility as prognosis marker and organized these deregulated T-UCRs by tumor types, recognizing the biological heterogeneity of tumors derived from different cells. 

T-UCRs and leukemia

The first association between UCRs and cancer, in 2007, included patients with chronic lymphocytic leukemia (CLL), and a panel composed of 19 UCRs was able to differentiate cancer from its non-tumor counterparts (Calin et al., 2007), including Uc.349/Uc.352. These T-UCRs are mapped on the chromosomal region 13q21.33-q22.2, a known familial CLL cancer-associated genomic region (Calin *et al*., 2007; Ng et al., 2007).

T-UCR involvement in CLL sensitivity to therapeutic agents was also evaluated, including analysis of these molecules after exposure to CpG-ODN, a toll-like receptor 9 agonist. All T-UCR expressions were screened in six primary CLL cases treated with CpG-ODN for 18 h, and Uc.70/Uc.414 were significantly down-regulated, confirmed in independent 12 CLL cases. These two T-UCRs were also previously associated with CLL (Calin et al., 2007). With data from a larger cohort, including more 67 cases and RNAseq data of 296 CLL from the International Cancer Genome Consortium project (Puente et al., 2015), the Uc.70 was highlighted as a potential prognosis marker in CLL (Bomben et al., 2019). 

Uc.70 is mapped on intronic region of the ARHGAP15 gene and overlaps with several sense and antisense transcripts, being the AC092652·2-202 suggested as the main transcript including uc.70 sequence. Uc.70/AC092652·2-202 transcripts were found to significantly predict time to treatment, which was significantly longer in patients with low expression, associated with poor prognosis (Bomben et al., 2019).

In pediatric acute lymphoblastic leukemia (ALL), the impact of T-UCRs associated with biological features and prognosis is not clear. T-ALL is usually more associated with poor prognosis when compared to B-ALL. Uc.112 was found more expressed in this group of patients, but, considering only B-ALL, Uc.112 was highly expressed in hyperdiploidy patients, a group considered as low risk of recurrence among B-ALL (das Chagas et al., 2021). 

Colon cancer

A distinct T-UCR signature in colorectal carcinoma (CRC) was also described in the first association of T-UCRs in cancer (Calin et al., 2007) and it includes 59 up- and two down-regulated molecules. For example, Uc.29, Uc.112, Uc.206, Uc.388, and Uc.399 are non-exonic UCRs with the most significant high expression in CRC (Calin *et al*., 2007). Uc.388 expression had opposite trend in a different CRC cohort (Sana et al., 2012), where down expression was found in 54 CRC tumor tissue compared to 15 samples of the adjacent unaffected tissue (Sana *et al*., 2012). Focused on T-UCRs with prognosis association, low Uc.388 expression was associated with the distal location metastasis (Sana *et al*., 2012). 

Uc.73 deregulation was also controversial; it was found down-regulated by Sana et al. (2012), and was one of the most up-regulated T-UCRs in colon cancer (Calin et al., 2007). Additionally, silencing of this RNA reduced proliferation and increased apoptosis levels, in concordance with a more oncogenic role in CRC cells (Calin *et al*., 2007). 

Also, with a potential oncogenic role and poor prognosis association, Uc.338 was found up-regulated in CRC tumor samples, and its expression was associated with larger tumor size, deeper invasion, and increased lymph node metastasis (Zhang et al., 2018). 

Related to chemotherapy resistance in CRC, Uc.160, Uc.283, and Uc.346 expression levels were significantly lower in 5-fluorouracil-resistant HT-29 cells than untreated cells. Uc.283 and Uc.346 expression were also reduced in oxaliplatin-resistant cells (Kottorou et al., 2020). 

The transcriptional deregulation of T-UCRs has been attributed to altered DNA methylation profile of the promoters, as down-regulation of Uc.160, Uc.283, and Uc.346 in colon cancer cells due to specific CpG island hypermethylation, reversed by induced hypomethylation (Lujambio et al., 2010) and both expression and methylation analysis may be investigated as useful biomarkers.

In tissue samples, the methylation levels of Uc.160, Uc.283, and Uc.346 are higher in CRC compared to adjacent non-tumor, followed by expression levels in an inverse pattern (Kottorou et al., 2018). Additionally, these three T-UCR methylation levels gradually increase from hyperplastic polyps to adenomas and *in situ* carcinomas; and a gradual decrease from in situ carcinoma to infiltrative/metastatic carcinomas occurs. Furthermore, higher Uc.160 and Uc.283 methylation were associated with better overall survival (Kottorou *et al*., 2020). 

Most highlighted T-UCRs were described in deregulation expression studies and results with polymorphism are limited, mainly because these regions are known to have low density of SNPs, however, looking for mutation and polymorphisms in UC genome regions, sequence abnormalities in 11 UCRs from 28 randomly selected ones were found. Among these mutations, six were found only in cancer patients - two in CLL and four in CRC samples. For example, considering potential role, a substitution in Uc.276 was predicted to change a miR-214 bind site, a microRNA known to be overexpressed in solid tumors (Wojcik et al., 2010). But, even with these descriptions, a clear association of SNPs as risk or protection markers are not found.

Liver cancer

The described hepatocellular carcinoma (HCC) signature originally included 8 T-UCRs. Three up-regulated: Uc.20, Uc.252, Uc.402 and five down-regulated: Uc.23, Uc.27, Uc.198, Uc.274, Uc.396 (Calin et al., 2007).

Another important T-UCR screening in HCC described 56 molecules aberrantly expressed in Hep-G2 cells compared with non-malignant hepatocytes. Among these, the most remarkable change was the high expression of Uc.338 in HCC cells. In a close genome region, Uc.339 is overexpressed in HCC cells and HCC-derived exosomes, thus contributing to a pro-tumoral HCC microenvironment (Kogure et al., 2013).

Breast cancer

In breast cancer (BC), a global screening for all 481 T-UCRs was performed using TCGA data (Pereira Zambalde et al., 2021). More than 60% were associated with at least one clinical feature that is important in BC; among them, 43% were associated with molecular subtypes, 36% with estrogen-receptor positivity, 17% with HER2 expression, 12% with stage, and 10% with overall survival (Pereira Zambalde *et al*., 2021). Furthermore, Uc.147 (or lnc-uc.147) was found highly expressed in luminal A and B patients, and for luminal A, up-regulation was associated with worse overall survival (Pereira Zambalde *et al*., 2021). The overexpression Uc.63 is also associated with poor prognosis in luminal A BC patients (Marini et al., 2017).

The association of 12 T-UCRs with clinical features was also analyzed in depth (Zambalde et al., 2022). Uc.84 was related to the HER2+ and low expression found in metastatic tumors, while Uc.376 was associated with ER+, PR+, and HER2+. The potential utility of T-UCRs as biomarkers was suggested. For example, a panel with Uc.147, Uc.271, and Uc.427 distinguished luminal A from triple-negative patients with an Area Under the Curve (AUC) of 0.95 (Zambalde *et al*., 2022). 

Despite the significant under-representation of single-nucleotide polymorphisms (SNPs) in UCRs, SNPs in these regions may be important in cancer patients. In BC, cancer-risk associated SNPs were highlighted in the uc.184, uc.313, uc.140, and uc.353 (Yang et al., 2008; Suvanto et al., 2020). But analysis of SNPs mapped in seven UCRs (uc.51, uc.82, uc.133, uc.140, uc.302, uc.353, and uc.368) failed to find an association in the Chinese population (Shen et al., 2011).

Lung cancer

Uc.61, Uc.83, Uc.280, Uc.338, and Uc.339 were found up-regulated in lung cancer (LC) tissues (Vannini et al., 2017; Tian and Feng, 2018; Liu et al., 2020; Vannini *et al*., 2022). Related to clinical features, high Uc.63 expression was associated with tumor stage and poor prognosis, while the Uc.280 expression was associated with patient age (Liu *et al*., 2020). 

Uc.338 has an important biomarker potential. The increased expression was associated with TNM stage, metastasis, and shorter overall survival and disease-free survival in non-small cell lung cancer, recognized as an independent risk factor (Tian and Feng, 2018). Uc.339 expression was also associated with poor survival (Vannini et al., 2017). These T-UCRs are mapped to just over 200,000 nucleotides away. Uc.338 in an intronic/exonic portion of the *PCBP2* gene and Uc.339 is an intergenic T-UCR but potential co-regulation of these molecules were not investigated previously.

An important down-regulated T-UCR described in lung carcinoma is Uc.454. The low expression was also correlated with higher tumor burden and advanced TNM stage. Additionally, the expression was associated with lymph node metastasis, tumor size, and stages, with the low expression being a potential poor prognosis marker (Zhou et al., 2018b). 

Prostate cancer

A first study that evaluated T-UCRs in prostate cancer (PC) included an analysis of all 481 T-UCRs in 57 PC tissues and seven non-tumor prostate samples, further cell line samples treated with epigenetic drugs, and synthetic androgen (Hudson et al., 2013). Many T-UCRs were found deregulated in PC patients, including Uc.106, Uc.477, Uc.363, Uc.454, and also T-UCRs responsive to drugs, such as Uc.287 induced by androgen and Uc.283 by combined 5-Aza 20 deoxycytidine and trichostatin treatment (Hudson *et al*., 2013). 

Analyzing 26 representative T-UCRs previously described (Hudson et al., 2013), Uc.63 was found increased in PC tissues. Also, in patients’ serum treated with docetaxel, Uc.63 expression was high in resistance compared to sensitive patients and associated with overall survival (Sekino et al., 2017). 

The 26 representative T-UCRs described by Hudson et al. (2013) were also evaluated by Goto et al. (2016) confirming down-regulation of 14 regions (Uc.73, Uc.118, Uc.158, Uc.241, Uc.244, Uc.249, Uc.252, Uc.261, Uc.282, Uc.346, Uc.359, Uc.389, Uc.390, and Uc.416) (Goto *et al*., 2016). Restored by DNA demethylation, Uc.158, Uc.241, and Uc.346 were highlighted, reinforcing the previous demonstration of Uc.241 induced expression in response to the 5-Aza-dC treatment (Hudson *et al*., 2013; Goto *et al*., 2016).

Genetic SNPs in UC regions were also studied in PC. Analyzing 14 SNPs in three cohorts of prostate cancer patients, rs8004379 in Uc.368 was associated with recurrence in localized disease. Additionally, rs8004379 was also associated with a decreased risk for prostate cancer-specific mortality (Bao et al., 2016).

Cervical and other gynecological cancers

Uc.338 is highly expressed in cervical cancer (CC) and associated with lymph node metastasis. Additionally, Uc.189 expression was evaluated in gynecological cancers, including 116 cervical squamous cell carcinomas, 98 endometrial adenocarcinomas, 29 ovarian cystoadenocarcinomas, and corresponding normal tissues. Uc.189 was found highly expressed in more than 70% of samples patients analyzed, and overexpression predicted poor prognosis in squamous cell and endometrial adenocarcinomas (Li et al., 2017; Wang et al., 2017).

Gastric cancer 

Uc.160 has been associated with GC, being found down-regulated in adenoma and GC tissues (Honma et al., 2017; Pang et al., 2018) and affected by hyper DNA methylation (Honma *et al*., 2017). Uc.416 was overexpressed in gastric cancer (GC) (Goto et al., 2016).

The oncogenic Uc.63 was also highlighted in GC. High expression of Uc.63 was found in GC tissues and associated with advanced stage and a tendency to show diffuse-type histology (Sakamoto et al., 2020).

Bladder cancer

Genome-wide profiling, including all T-UCRs, was evaluated in bladder cancer (BlC) and highlighted Uc.8 as being the most up-regulated and Uc.217 the most down-regulated ones (Olivieri et al., 2016). Uc.8 had the highest up-regulation compared to normal bladder epithelium but had a significantly low expression compared to pericancerous bladder tissues, being associated with grading and staging of bladder cancer. Interestingly, there is a simultaneous presence of Uc.8 in the cytoplasm/nucleus in low-grade patient samples and more cytoplasmic localization in high-grade samples (Terreri et al., 2021).

Like prostate cancer, Uc.63 was also associated with drug resistance in BlC (Sekino et al., 2019). Uc.63 was found to be highly expressed in urothelial carcinoma compared to non-tumor bladder tissues and 15 types of normal tissue (Sekino *et al*., 2019). 

Neurological cancer

The first study about T-UCRs in neurological cancers investigated all 481 regions in 34 high-risk neuroblastoma patients (Scaruffi et al., 2009). Focused on predicting outcomes, the authors described that 54 of the detectable T-UCRs showed a differential expression between the long and short survival patients with at least 15 up-regulated T-UCRs are needed to discriminate survival groups (Scaruffi *et al*., 2009). Additionally, 9 T-UCR expression (Uc.209, Uc.271, Uc.312, Uc.330, Uc.371, Uc.411, Uc.421, Uc.435, Uc.452) expression are inversely correlated with 5 complementary microRNA (miR-33b*, miR-383, miR-877*, miR-548d-5p, miR-939) (Scaruffi *et al*., 2009).

Profiles of T-UCRs in representative neuroblastoma tumors and a signature of seven T-UCRs (uc.347, uc.350, uc.279, uc.460, uc.379, uc.446, uc.364) were found highly expressed in highly aggressive MYCN-amplified tumors compared to MYCN-non-amplified samples (Mestdagh et al., 2010).

Another neurological tumor described with T-UCRs deregulation is glioma. Uc.160 is the epigenetic silenced in this type of cancer and an independent prognostic factor associated with better overall survival in lower-grade gliomas (Soler et al., 2022). High expression of Uc.283 was also evidenced in glioma (Galasso et al., 2014).

Others

Other tumor types also highlighted the importance of T-UCRs and were potentially helpful as diagnostic/prognosis markers. In pancreatic cancer, a screening of all 481 T-UCRs was evaluated in cancer specimens, pancreatic cancer cell lines, during experimental pancreatic desmoplasia, and mice models. T-UCRs were differentially expressed in 14% of cell lines, in 57% of human tumors, 25% in pancreatic desmoplasia, and 29% of a transgenic mouse model. In the three human data sets, Uc.190, Uc.233, and Uc.270 were highly expressed (Jiang et al., 2016).

In renal cell carcinoma, Uc.416 was found highly expressed compared to normal kidney tissues (Sekino et al., 2018), and Uc.189 expression was significantly higher in human esophageal squamous cell carcinoma (ESCC). The high level was significantly correlated with invasion, advanced clinical stage, lymph node metastasis, and poor prognosis (Guo et al., 2017). 

## Conclusion

**Figure 4 -:**
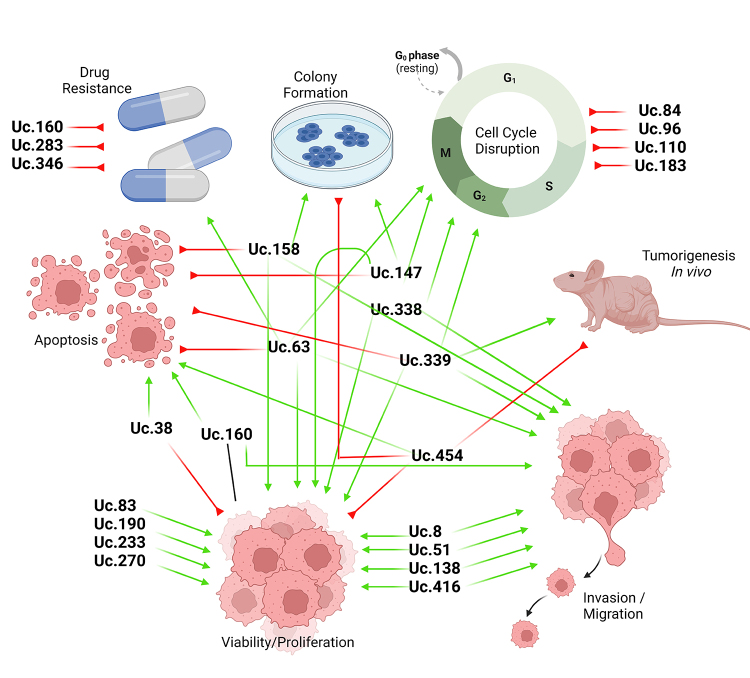
Transcribed ultraconserved regions (T-UCRs) associated with cancer cell processes. They mostly affect cell proliferation, migration/invasion and apoptosis in distinct tumor types. Green arrows meaning promotion and red arrows meaning suppression of the mechanisms. Created with BioRender.com

The great number of studies describing T-UCRs associated with several features in diverse tumor types emphasize the important roles of these molecules in cancer cells, mainly in proliferation, apoptosis, and migration/invasion (Figure 4). But, further investigation about these molecules must be performed.

Most of the available papers in this field describe T-UCRs with deregulated expression in tumor cells. Out of 481 T-UCRs, we found that 23 molecules are associated with tumorigenesis processes, 297 present differential expression in samples of patients with cancer, and 12 of them with clearer potential utility as prognosis markers (Table 1). 

**Table 1 -:** T-UCRs with potential utility as prognosis marker in human cancers.

T-UCRs	Tumor type	Association	Number of patients	Prognosis relevance	References
Uc.63	Luminal A BC LC PC GC	worse overall survival tumor stage drug resistance overall survival advanced stage	354 50 79 40	poor poor poor poor	Marini et al., 2017 Liu et al., 2020 Sekino et al., 2017 Sakamoto et al., 2020
Uc.70	CLL	short time to treatment	67	good	Bomben et al., 2019
Uc.84	BC	less distal metastasis	827	good	Zambalde et al., 2022
Uc.112	ALL	T-cell ALL	62	poor	das Chagas et al., 2021
Uc.147	Luminal A BC	worse overall survival	364	poor	Pereira Zambalde et al., 2021
Uc.160	CRC	better overall survival	137	good	Kottorou et al., 2020
Uc.189	CC ES	tumor size TNM stage distant metastasis invasion advanced clinical stage lymph node metastasis	116 152	poor poor	Wang et al., 2017 Guo et al., 2017
Uc.283	CRC	better overall survival	137	good	Kottorou et al., 2020
Uc.338	CRC LC CC	larger tumor size deeper invasion increased lymph node metastasis TNM stage, metastasis shorter overall survival and disease-free survival lymph node metastasis	100 185 40	poor poor poor	Zhang et al., 2018 Tian and Feng 2018 Li et al., 2017
Uc.339	LC	poor survival	30	poor	Vannini et al., 2017
Uc.388	CRC	less distal metastasis	54	good	Sana et al., 2012
Uc.454	LC	low TNM stage and tumor size less lymph node metastasis	98	good	Zhou et al., 2018b

One challenge to better investigate these molecules is the little information about the molecular details of the transcript. For example, only 4% of T-UCRs present detailed information about the molecule, including complete sequence and/or cell localization (Pereira Zambalde et al., 2020). Additionally, the studies reviewed herein provide new possibilities for developing diagnostic and prognostic markers, although this insight has not been deeply analyzed yet. In other words, even with the increase of studies focusing on T-UCRs and their potential in molecular marker utility, real application in clinical contexts or strategies to target T-UCRs in clinical trials have not been explored yet.

T-UCRs were associated with different hallmarks and showed great potential as biomarkers in many tumor types. For example, the Uc.63 high expression was found in several tumor types and associated with poor prognosis and also to the highlighted important role of Uc.63 in sustaining proliferative signaling, inducing invasion and migration, repressing apoptosis, and association with drug resistance. On the other hand, some T-UCRs have their expression and role more restricted to single tumor types.

Based on this review, T-UCRs are deregulated in cancer and are highlighted as important molecules in tumor cell networks. Furthermore, T-UCRs have potential as diagnostic/prognostic markers, although they may be better investigated for translational medicine. 
